# Propylthiouracil-Induced Anti-Neutrophil Cytoplasmic Antibody (ANCA)-Associated Vasculitis Presenting with Multiple Sterile Abscesses, Mononeuritis Multiplex, and Splenic Vein Thrombosis: A Case Report and Literature Review

**DOI:** 10.7759/cureus.61229

**Published:** 2024-05-28

**Authors:** Ioannis Karageorgiou, Ashbina Pokharel, Ashbita Pokharel, Ewelina Niedzialkowska, Judith Bateman

**Affiliations:** 1 Internal Medicine, William Beaumont University Hospital, Royal Oak, USA; 2 Pathology, William Beaumont University Hospital, Royal Oak, USA; 3 Rheumatology, William Beaumont University Hospital, Royal Oak, USA

**Keywords:** splenic venous thrombosis, autoantibodies, drug-induced abnormalities, propylthiouracil, anti-neutrophil cytoplasmic antibody-associated vasculitis

## Abstract

Anti-neutrophil cytoplasmic antibody-associated vasculitis (AAV) represents a rare group of disorders, that traditionally includes diseases like granulomatosis with polyangiitis (GPA), eosinophilic granulomatosis with polyangiitis (EGPA), and microscopic polyangiitis (MPA). However, AAV can also be triggered by medications such as propylthiouracil (PTU). This article focuses on the subset of drug-induced AAV. We examine how certain medications, notably PTU, can provoke an AAV response, detailing the pathophysiological mechanisms and clinical implications. A 72-year-old female being treated with PTU presented with bilateral hand abscesses, generalized weakness, and frequent falls. Despite initial treatments, her condition worsened, prompting consideration of AAV secondary to PTU. Following appropriate diagnostic procedures and initiation of treatment, including steroids, heparin, and rituximab, the patient showed significant improvement. PTU-induced AAV is a serious, albeit rare, side effect characterized by anti-neutrophil cytoplasmic autoantibodies, with the potential for varied organ involvement and generally a better prognosis than primary AAV. The atypical presentation in this case underscores the importance of clinician vigilance and awareness, ensuring timely diagnosis and appropriate management of this complex condition.

## Introduction

Anti-neutrophil cytoplasmic antibody-associated vasculitis (AAV) is a group of rare disorders that affect small-to-medium-sized vessels, namely arterioles, venules, and capillaries. The three main diseases in this category are granulomatosis with polyangiitis (GPA), eosinophilic granulomatosis with polyangiitis (EGPA), and microscopic polyangiitis (MPA) [1]. These diseases have been described as separate clinical entities since the discovery of anti-neutrophil cytoplasmic antibodies (ANCAs) in 1982 [2]. However, it was not until more recently that they were distinguished from one another. MPA was the last of the three to be described as a separate clinical entity, distinguished from polyarteritis nodosa in 1994 at the Chapel Hill Consensus Conference (CHCC) [3]. Even though ANCA autoantibodies have been identified as a key factor in pathogenicity, leading to tissue damage through a cascade of events involving innate and adaptive immune responses [2], questions remain as to how and why these autoantibodies develop. Many genetic and environmental factors that contribute to the development of the disease have been found [2,4]. Characteristic examples include major histocompatibility complex (MHC) genes such as HLA-DP and HLA-DQ and non-MHC genes, including PTPN22, SERPINA1, and PRT3 [2]. Environmental factors associated with the development of AAV include exposure to silica, smoking, geographic location, and various infections, such as *Staphylococcus aureus* [2,4]. In addition, many drugs have known associations with the development of ANCA autoantibodies, and AAV [5]. These include antibiotics (cefotaxime, minocycline), antithyroid drugs (propylthiouracil, methimazole, carbimazole), anti-tumor necrosis factor agents (infliximab, etanercept), psychoactive agents (clozapine, thioridazine) and various other drugs (allopurinol, hydralazine, phenytoin, sulfasalazine). The development of vasculitis as a result of exposure to a drug is described as a separate clinical entity, usually considered an adverse drug reaction [2,5]. One of the most well-known and by far the most reported drug-induced AAV is caused by the antithyroid drug propylthiouracil (PTU) [6-9]. It was first described as a case report in 1992 by Stankus and Johnson [10]. Since then, over 100 cases of PTU-induced AAV have been described in the literature [5], including many retrospective reviews. This disease usually presents in middle-aged females with a history of Graves’ disease that is being treated with PTU for varying periods [6,11,12]. Disease severity is variable, from mild systemic symptoms such as fever, fatigue, and arthralgias, to organ-threatening and life-threatening complications most commonly affecting the renal and pulmonary systems, including pauci-immune rapidly progressive glomerulonephritis, diffuse alveolar hemorrhage, and death [7,13,14]. With the above facts in mind, our case report is unique because the presentation of the disease was atypical; No similar cases were found in the literature review that was conducted and presented in this paper. Our case describes an elderly female with high anti-myeloperoxidase (anti-MPO) and perinuclear anti-neutrophil cytoplasmic antibody (p-ANCA) titers, diagnosed with PTU-induced AAV. Her unusual presentation with a combination of cutaneous, vascular, and neurologic manifestations makes this case a compelling addition to the literature, providing valuable insights for future clinicians encountering similar presentations.

This case was previously presented as an abstract at the American College of Physicians 2023 National Abstract Competition, held in San Diego, CA, on April 28, 2023.

Literature review

We performed a narrative literature review to explore drug-induced ANCA-associated vasculitis, focusing on PTU-induced cases. Our medical librarians assisted with a targeted PubMed search, limited to articles published in English over the last 15 years. Using search terms such as 'drug-induced AAV' and 'PTU-induced AAV,' we initially identified 40 relevant papers. We prioritized studies specifically reporting on PTU-induced vasculitis, including 17 PTU-specific articles, four on thyroid drug-associated vasculitis, and case reports involving methimazole, carbimazole, and concurrent PTU and methimazole use. Additionally, we reviewed 11 articles on treatment strategies, and three on drug-associated vasculitis, and further analyzed six additional case reports and six articles on the prevalence, pathophysiology, and management of AAV. This narrative review, though not systematic, aimed to provide a comprehensive understanding of PTU-induced AAV to support our case report and discussion.

## Case presentation

A 72-year-old Caucasian female with a history of hyperthyroidism presented with painful bilateral hands, generalized weakness, and frequent falls. The patient was taking PTU 50 mg daily for one and half years until three weeks before presenting. Her endocrinologist suspected Graves’ disease as the cause of her hyperthyroidism, although she never demonstrated positive thyroid autoantibodies. Due to the over-blocking of thyroid hormone production, indicated by a thyroid-stimulating hormone (TSH) level of 14.61 µIU/mL (normal range: 0.4-4.0 µIU/mL) three weeks prior, her medication was discontinued. Upon evaluation by our hospital's endocrinology team, her TSH level had normalized to 2.82 µIU/mL.

**Figure 1:**
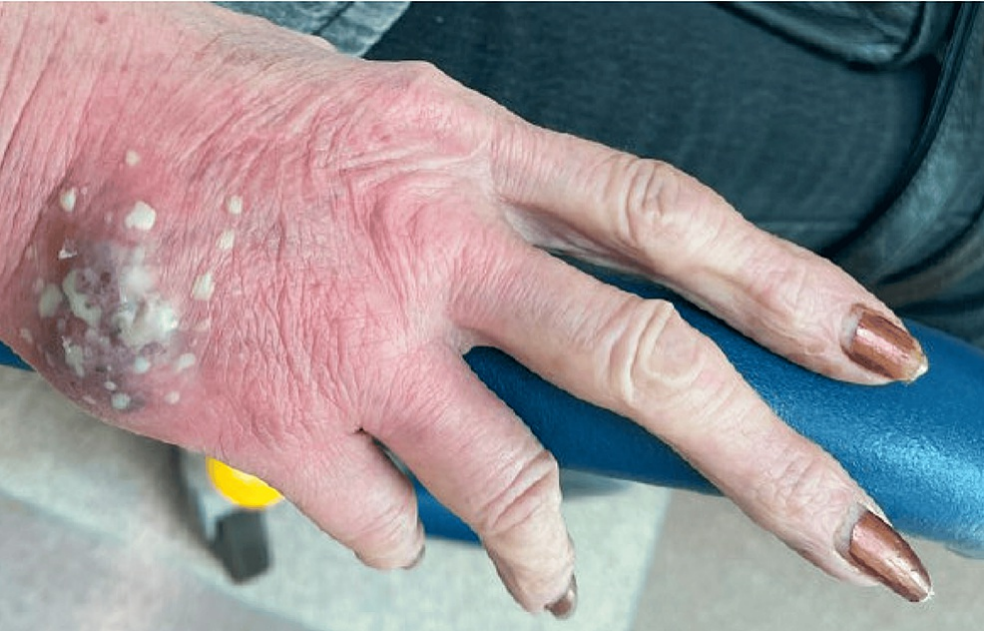
Abscess formation on the lateral side of the dorsum of the right hand.

Examination revealed erythematous and purple fluctuant tender masses on the dorsum of both the right and left hands (Figure 1). X-ray of both hands did not reveal any abnormalities.

Her laboratory results on presentation are shown in Table 1. Despite treatment with intravenous ceftriaxone and vancomycin for suspected bacterial abscesses, the patient developed new necrotic appearing fluctuant masses in multiple areas of her body and started having increased oxygen requirements. She also developed a right foot drop, which was not present during the initial physical examination.

**Table 1:** Laboratory results on presentation. MCV: Mean corpuscular volume; MCH: Mean corpuscular hemoglobin; RBC: Red blood cells.

Variable	Result	Reference range
White blood cell count	15.4 bil/L	3.3-10.7 bil/L
Neutrophil count	12.7 bil/L	1.6-7.2 bil/L
Hemoglobin	9.3 g/dL	12.1-15.0 g/dL
MCV	90 fL	80-100 fL
MCH	28 pg	28-33 pg
Platelets	454 bil/L	150-400 bil/L
Blood urea nitrogen	21 mg/dL	7-25 mg/dL
Creatinine	0.92 mg/dL	0.60-1.40 mg/dL
Total bilirubin	0.7 mg/dL	0.3-1.2 mg/dL
C-reactive protein	207 mg/dL	0.0-0.8 mg/dL
Erythrocyte sedimentation rate	>130 mm/hr	0-18 mm/hr
Urinalysis protein	100 mg/dL	0 mg/dL
Urinalysis blood	1+	0
Urinalysis RBC	0-2/hpf	0–2/hpf
Urine protein to creatinine ratio	1.49	0.00-0.20

She underwent incision and drainage of several abscesses and escalation of antibiotics to meropenem, vancomycin, and valacyclovir. Blood, urine, and wound cultures were all negative. Extensive infectious, and initial rheumatologic workup with serum antinuclear antibody (ANA) and rheumatoid factor (RF; normal range: <1:160 for ANA, <15 IU/mL for RF, shown in Table 2) was negative.

**Table 2:** Infectious and rheumatologic workup. ANCA: Anti-neutrophilic cytoplasmic antibody; PR3: Proteinase 3; MPO: Myeloperoxidase; IgG: Immunoglobulin G; IgA: Immunoglobulin A; IgM: Immunoglobulin M; C3: Complement component 3; C4: Complement component 4; R: Receptor; HBV: Hepatitis B virus; HCV: Hepatitis C virus; HIV: Human immunodeficiency virus.

Variable	Result	Reference range
Antinuclear antibodies	Negative	Negative (<1:160)
Rheumatoid factor	<15 IU/mL	<15 IU/mL
Anti-neutrophilic cytoplasmic antibody (ANCA)	≥ 1:640	<1:20
ANCA pattern	P ANCA	n/a
ANCA-PR3	3 U	≤ 20 U
ANCA-MPO	41 U	≤ 20 U
Glomerular basement membrane antibody, IgG	3 U	0-20 U
Total IgG	977 mg/dL	550-1,650 mg/dL
Total IgA	170 mg/dL	70-365 mg/dL
Total IgM	292 mg/dL	30-263 mg/dL
Complement C3	128 mg/dL	82-193 mg/dL
Complement C4	33 mg/dL	10-43 mg/dL
Cryoglobulins serum	negative	negative
Acetylcholine R blocking antibody	5%	0-26%
Acetylcholine R binding antibody	0.0 nmol/L	0.0-0.4 nmol/L
Acetylcholine R modulating antibody	0%	≤45%
Hepatitis B surface antigen	nonreactive	nonreactive
Hepatitis B core antibody, IgM	nonreactive	nonreactive
Hepatitis B core antibody, total	nonreactive	nonreactive
Anti-HCV antibody	nonreactive	nonreactive
HIV-1 p24 antigen/HIV1/2 antibody screen	nonreactive	nonreactive
Cryptococcal serum antigen	negative	negative
Aspergillus galactomannan antigen, serum	0.03	0.00-0.50
Serum (1→3)-β-D-Glucan (Fungitell®)	<31 pg/mL	≤59 pg/mL
Histoplasma antigen, Urine	None deteted	negative

An MRI of her hand revealed “diffuse soft tissue and muscle edema and skin ulceration” which was non-specific. A CT angiogram of the chest and abdomen showed left upper and left lower lobe segmental branch pulmonary emboli and splenic vein thrombosis. A repeat chest X-ray (CXR) showed worsening diffuse bilateral airspace disease. A 2D echocardiogram and transesophageal echocardiogram showed an ejection fraction of 65% (normal range: 55-70%) and no vegetation. Electromyography (EMG) indicated absent right peroneal motor nerve conduction. Further rheumatologic workup (shown in Table 2) revealed elevated perinuclear anti-neutrophil cytoplasmic antibodies (p-ANCA >1:640, normal range: <1:20), and elevated anti-myeloperoxidase antibodies (MPO 41 U, normal range: <20 U). Acetylcholine receptor antibodies were found to be negative. Skin biopsy showed subcorneal pustules filled with neutrophils and immunofluorescence demonstrated no evidence of immune-bullous dermatitis. Vasculitis was not observed. Sural nerve and muscle biopsies were obtained. Due to the constellation of findings of multiple sterile abscesses, elevated p-ANCA, pulmonary embolism, splenic vein thrombosis, hematuria, proteinuria, and left foot drop suggestive of mononeuritis multiplex, in combination with the patient’s clinical deterioration despite being on broad-spectrum antibiotic therapy, an autoimmune condition was starting to become increasingly more likely. Considering the patient’s strong p-ANCA positivity and her history of PTU use, AAV secondary to PTU was considered. While hematuria and proteinuria suggested renal involvement, the patient did not show clinical or laboratory signs of renal impairment, such as elevated creatinine or decreased glomerular filtration rate. The likely cause was mild glomerular involvement from PTU-induced vasculitis, which did not warrant a renal biopsy. The patient was started on intravenous methylprednisolone 250 mg every six hours for three days. Intravenous heparin was used to treat thromboembolism.

**Figure 2:**
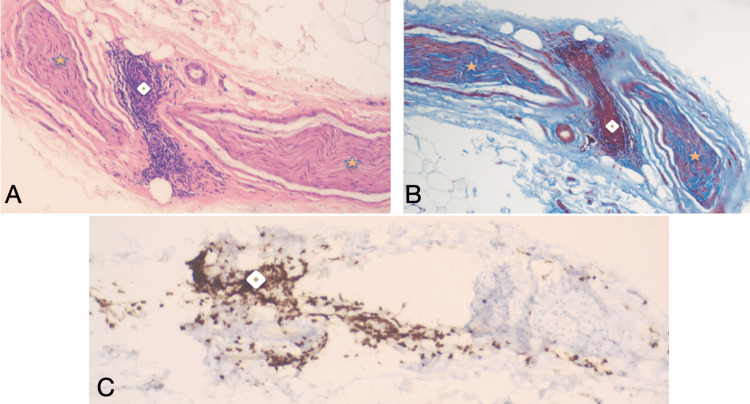
Sural nerve biopsy. A blood vessel with lymphocyte infiltration (diamond) is evident, and the cells are identified as CD3+ T-cells. Nerve fibers are visible on either side of the blood vessel (yellow asterisk). These findings support a diagnosis of small vessel vasculitis in the sural nerve fibers. A: Hematoxylin and eosin stain, 10X magnification; B: Masson trichrome stain, 10X magnification; C: CD3 staining, 10X magnification.

The histopathological examination of the sural nerve biopsy revealed several noteworthy findings: hematoxylin and eosin (H&E) stained sections showed areas with increased T cell lymphocytic infiltrate, which was further confirmed by CD3 staining, highlighting the elevated number of lymphocytes predominantly in the perineural tissues. Additionally, rare foci of vasculitis were observed within the sural nerve. Masson trichrome stain corroborated the presence of vasculitis (Figure 2).

Rituximab 375 mg/m2 weekly for four weeks was added, and the intravenous methylprednisolone was transitioned to prednisone 60 mg daily. Dapsone was used for *Pneumocystis jirovecii *prophylaxis. The patient had a good clinical response to treatment, her skin lesions began to heal and no new lesions appeared. Respiratory status improved, and a repeat CXR showed an improvement in airspace opacities, and the patient was weaned off oxygen. The patient was discharged after a prolonged hospital stay, continuing on prednisone 60 mg daily with two more infusions of rituximab remaining. The patient was readmitted two months post-discharge due to a non-ST-elevation myocardial infarction (NSTEMI). In the meantime, she had completed four weeks of rituximab infusions. Unfortunately, her steroid taper was delayed at an extended care facility, but after several months she was tapered off steroids entirely. Her skin lesions healed completely within weeks of treatment initiation, and her strength in the lower extremities improved. The patient has remained in remission from vasculitis for 19 months.

## Discussion

To the best of our knowledge, no other cases in the literature report a patient treated with PTU for hyperthyroidism presenting with multiple sterile abscesses, bilateral segmental pulmonary embolism, splenic vein thrombosis, mononeuritis multiplex, and high anti-MPO and p-ANCA titers, diagnosed with PTU-induced AAV. The atypical character of this case is attributed to the patient’s age and clinical presentation with cutaneous, vascular, and neurologic complications, which are far less common than the renal and pulmonary complications usually seen in this type of vasculitis [6,7,15,16].

Some case reports describe AAV presenting with venous thromboembolism (VTE) [17], and one case report described cerebral vasculitis as a manifestation of the disease [13]. Even though ANCA autoantibodies are implicated in primary AAV, different mechanisms likely contribute to PTU-induced AAV [2]. This is evident from the variability in antibodies observed; primary AAV typically shows either p-ANCA or c-ANCA, while PTU-induced AAV is associated with antibodies against MPO, PR3, beta-2 glycoprotein, lactoferrin, cathepsin G, neutrophil elastase, and azurocidin [5,6,14]. In our case, no other autoantibodies were identified. Notably, ANCA levels in drug-induced AAV do not consistently correlate with disease activity, and many patients remain asymptomatic despite being ANCA-positive [5,6,8]. Interestingly, MPO antibody avidity may be a better marker for disease activity, as it decreases faster than antibody titers during remission [5]. In primary AAV, a vicious cycle between ANCA formation and neutrophil activation causes vascular damage, with the exact trigger being difficult to identify (genetic and environmental factors mentioned earlier). In PTU-induced AAV, although the mechanism is unclear, several theories exist. PTU metabolites can mask DNase I recognition sites in DNA extruded in neutrophil extracellular traps (NETs) [2]. DNase I cleaves NETs, naturally present in our immune system. Failure to degrade NETs, rich in MPO, leads to autoantibody development as these molecules act as autoantigens [2]. A similar mechanism is seen in SLE and hydralazine drug-induced SLE [2]. Other mechanisms include the similarity of the thyroid peroxidase molecule to MPO, as well as the molecular modification of MPO induced by PTU (a change in the protein structure surrounding the heme iron from a rhombic to an axial form) which is thought to contribute to the breakdown of tolerance to MPO [2,5]. PTU-induced AAV is mostly observed in younger patients (ages 19-69, average 39.4±15.3 years) and is more common in females with Graves’ disease [5,6,11]. This distribution likely reflects the demographics of those treated with PTU [5]. In contrast, primary AAV is more common in older adults (ages 60-70) and affects males and females equally [5,18]. Ethnic variations exist, with higher PR3 positivity in Europeans and higher MPO positivity in Japanese populations [4,5]. Our patient, being elderly and without Graves’ disease, presents an atypical case. PTU-induced AAV has been well-documented, with vasculitis occurring in 0.47-0.71 per 10,000 patients with Graves’ disease, but ANCA positivity in 15-64% of PTU-treated patients, indicating that ANCA positivity does not always correlate with clinical disease. Vasculitis typically develops after a year of PTU treatment (median 36-42 months) but can appear sooner [5-7]. Long-term PTU use is a risk factor for AAV, necessitating vigilance for vasculitis symptoms in these patients [5]. Methimazole and carbimazole are alternative treatments for Graves’ disease with lower AAV incidence, making them suitable for patients who develop PTU-induced AAV [5,7-9].

The most affected systems in AAV include renal, pulmonary, and skin [6,7,15,16], with common symptoms being hematuria, proteinuria, hemoptysis, dyspnea, ulcers, purpura, and rash [7]. Less common symptoms include joint swelling, uveitis, abdominal pain, and multiple cranial nerve deficits [7], with VTE also reported [17]. Cutaneous manifestations can vary widely, with sterile pustules/abscesses being less common [16]. AAV and other autoimmune diseases are significant risk factors for VTE, with a 9.7% incidence in AAV patients, significantly higher than the general population [15]. Evidence suggests that inflammation is a key factor contributing to increased VTE risk in AAV patients by enhancing the hypercoagulable state and increasing endothelial damage [15]. Inflammatory mediators released by activated neutrophils promote a pro-coagulant state by inducing tissue factor expression and increasing endothelial damage, contributing to hypercoagulation even during remission [15]. The excessive activation of neutrophils by ANCA leads to the formation of NETs, which further damage endothelial cells, activate the complement system, and release tissue factor, initiating the extrinsic pathway of coagulation [15]. NETs, containing DNA fragments, activate factor XII and platelets, promoting thrombin formation and amplifying coagulation cascades, particularly the intrinsic pathway [19]. In PTU-induced AAV, NETs may play a central role by triggering ANCA production, contributing to both vasculitis and thrombosis. Studies indicate that in active AAV, NET formation is abundant and closely associated with thrombi, suggesting that NETs are significant drivers of hypercoagulability through activation of both intrinsic and extrinsic pathways [15,19]. AAV-related VTE is treated with standard anticoagulation therapies [17], and common VTE sites include deep veins, pulmonary embolism, and rare splanchnic thromboses [17].

Diagnosis of PTU-induced AAV typically involves histologic evidence of vasculitis, serum ANCA presence, and exposure to a causative drug like PTU. Common diagnostic specimens include renal or skin biopsies, with bronchoscopy used to detect diffuse alveolar hemorrhage [9,17]. In our case, a sural nerve biopsy confirmed vasculitis as skin biopsies were inconclusive, and renal involvement was mild at presentation. Although there are no specific randomized trials for drug-induced AAV treatments, it generally has a better prognosis than primary AAV, with less aggressive disease, lower end-stage renal disease (ESRD) risk, and fewer relapses [5-8]. The primary treatment step is discontinuing the causative drug without rechallenge [5,14]. Mild cases may not require further treatment, but severe cases often need immunosuppressive therapy similar to primary AAV, including steroids with cyclophosphamide or rituximab, followed by a taper [1,5,14,20]. Steroids can be tapered more quickly in drug-induced AAV, and maintenance therapy is usually unnecessary as relapses are rare without drug re-exposure [6-8,14]. Our patient received pulse dose steroids and rituximab induction due to severe lung and skin involvement. The steroid taper was completed in 20 weeks without maintenance therapy. Follow-up typically includes clinical and laboratory monitoring for vasculitis relapse and thyroid function assessment. Symptoms generally resolve shortly after PTU discontinuation and treatment initiation, with minimal cases of relapse or permanent organ damage [8]. Our patient remained euthyroid post-PTU discontinuation and did not require additional thyroid medication. Respiratory and cutaneous symptoms improved within a week of treatment. Despite an NSTEMI months later, her vasculitis remained stable. It is noted that AAV patients have a higher risk of cardiovascular events within six months of diagnosis [15].

## Conclusions

Overall, this case highlights the need for heightened vigilance for propylthiouracil (PTU)-induced anti-neutrophil cytoplasmic antibody-associated vasculitis (AAV), particularly in atypical presentations involving cutaneous, vascular, and neurologic symptoms. Early recognition and appropriate management are crucial to improving patient outcomes and preventing severe complications. Clinicians should consider discontinuing PTU and initiating immunosuppressive therapy promptly in suspected cases. Additionally, the successful treatment with steroids and rituximab highlights the importance of a personalized approach to managing drug-induced AAV. The patient’s favorable response to immunosuppressive therapy and subsequent remission reinforces the potential for positive outcomes when the condition is promptly and adequately addressed. This case contributes valuable insights to the existing literature, offering a reference point for future clinicians who may encounter similar presentations and aiding in the ongoing effort to optimize diagnostic and therapeutic strategies for drug-induced AAV.
